# Role of FDG PET/CT in monitoring treatment response in patients with invasive fungal infections

**DOI:** 10.1007/s00259-018-4192-z

**Published:** 2018-10-21

**Authors:** Alfred O. Ankrah, Lambert F. R. Span, Hans C. Klein, Pim A. de Jong, Rudi A. J. O. Dierckx, Thomas C. Kwee, Mike M. Sathekge, Andor W. J. M. Glaudemans

**Affiliations:** 1 Medical Imaging Center, Departments of Nuclear Medicine and Molecular Imaging and Radiology, University of Groningen, University Medical Center Groningen, Groningen, The Netherlands; 2 Department of Nuclear Medicine, Steve Biko Academic Hospital, University of Pretoria, Pretoria, South Africa; 3 Nuclear Medicine Unit, National Centre for Radiotherapy and Nuclear Medicine, Korle Bu Teaching Hospital, Accra, Ghana; 4 Department of Internal Medicine, Division of Hematology, University of Groningen, University Medical Center Groningen, Groningen, The Netherlands; 5 Department of Psychiatry, University of Groningen, University Medical Center Groningen, Groningen, The Netherlands; 6 Department of Radiology and Nuclear Medicine, University Medical Center Utrecht, and Utrecht University, Utrecht, The Netherlands

**Keywords:** Invasive fungal infections, Therapy monitoring, Metabolic parameters, FDG-PET/CT, Total lesion glycolysis, Antifungal therapy

## Abstract

**Introduction:**

Invasive fungal infections (IFIs) occur mostly in immunosuppressed patients and can be life-threatening. Inadequate treatment is associated with high morbidity and mortality. We examined the role of 2-fluorodeoxyglucose positron emission tomography integrated with CT (FDG-PET/CT) in monitoring IFIs and therapy decision-making, and evaluated the role of baseline metabolic parameters in predicting the metabolic response.

**Methods:**

All patients between October 2009 and March 2018, diagnosed with IFIs, treated with antifungal drugs, and who underwent FDG-PET/CT at baseline and at one or more timepoints during treatment were retrospectively included. The electronic patient files were reviewed for pathology, microbiology, and laboratory findings. All FDG-PET/CT scans were performed according to standardized European Association of Nuclear Medicine/EANM Research Limited (EANM/EARL) protocols. For each scan, the global total lesion glycolysis (TLG) and metabolic volume (MV), highest maximum standardized uptake value (SUVmax), and peak standardized uptake value (SUVpeak) were determined. The role of FDG-PET/CT on monitoring antifungal therapy was assessed by looking at the clinical decision made as result of the scan. Furthermore, the added value of the baseline metabolic parameters in predicting metabolic response to the antifungal treatment was evaluated.

**Results:**

Twenty-eight patients with in total 98 FDG-PET/CT scans were included with a mean age of 43 ± 22 years. FDG-PET/CT altered management in 14 out of the 28 patients (50%). At the final FDG-PET/CT scan, 19 (68%) had a complete metabolic response (CMR), seven a partial response and two patients were defined as having progressive disease. Using receiver operative analysis, the cut-off value, sensitivity, specificity, and significance for the baseline TLG and MV to discriminate patients with CMR were 160, 94%, 100%, *p* < 0.001 and 60, 84%, 75%, *p* = 0.001 respectively.

**Conclusion:**

FDG-PET/CT is useful in the monitoring of IFIs resulting in management therapy change in half of the patients. Baseline TLG and MV were found to be able to predict the metabolic response to antifungal treatment.

**Electronic supplementary material:**

The online version of this article (10.1007/s00259-018-4192-z) contains supplementary material, which is available to authorized users.

## Introduction

Invasive fungal infections (IFIs) often occur in immunosuppressed patients and can be life-threatening. Timely monitoring of the efficacy of antifungal drugs in patients with IFIs is crucial. There are only a few classes of antifungals available, and it is crucial to preserve the effectiveness of these agents [1]. Antifungal drugs are costly and frequently accompanied by severe and sometimes intolerable side-effects. Patients with IFIs are usually treated for extensive periods (months to sometimes even years), and the duration of treatment is not standardized. Inappropriate treatment with antifungal agents may potentially result in potential fungal disease and can induce resistant fungi, which increase morbidity or mortality [1–3]. Morbidity and mortality for IFIs vary considerably depending on the type of IFI and underlying disease predisposing to IFI [4, 5]. The mortality rates for the most common organisms causing IFIs are usually about 30% [5]. Inadequate treatment of IFIs may result in dissemination of the infection during immunosuppressive procedures such as intensive chemotherapy or stem-cell transplantation that are often used to treat underlying conditions associated with IFIs. It is therefore critical to determine in a timely manner whether the antifungal treatment regimen is adequate or whether modification of therapy is required. Imaging techniques provide a non-invasive method to determine treatment response and therefore can be used as treatment follow-up of IFIs. Anatomical imaging, particularly with (chest and abdominal) computed tomography (CT) but also with brain magnetic resonance imaging (MRI), is usually used for the management of IFIs [4, 6]. However, the anatomical changes associated with IFIs may persist for long periods, even after adequate treatment, thereby potentially delaying any further therapy that may be required for the underlying disease [2]. Functional imaging with 2-fluorodeoxyglucose positron emission tomography integrated with CT (FDG-PET/CT) has been found useful in the monitoring of IFIs in a relatively small numbers of studies and case reports available on this topic [2].

FDG-PET/CT has been used to monitor treatment in mainly oncological diseases but more recently also in infectious diseases [7]. In monitoring disease, FDG-PET/CT has the advantage of using metabolic indices which provide absolute quantification of the disease [8]. The metabolic indices have also been shown to have prognostic value in different diseases [9, 10]. Most widely used is the maximum standardized uptake value (SUVmax). Other indices include the mean standardized uptake value (SUVmean), peak standardized uptake value (SUVpeak), metabolic volume (MV) and total lesion glycolysis (TLG). Each of these parameters has its advantages and limitations, and there is no real agreement which is the best parameter to use. SUVmean, MV, and TLG utilize the uptake from the whole lesion rather than the highest voxel for SUVmax or the highest uptake in a 1-ml volume (SUVpeak), and may be more representative of disease burden in the lesion. TLG and MV have been evaluated in oncology and more recently in inflammatory processes [9–12]. TLG was found to be reproducible and highly correlated with other PET metrics in the assessment of the response of treatment [8]. To the best of our knowledge, metabolic parameters such as TLG and MV have not been investigated in infections.

In this study, we (1) examine the role of serial FDG-PET/CT in monitoring IFIs for therapy decision-making, and (2) evaluate the role of the baseline metabolic parameters in predicting the metabolic response to antifungal treatment.

## Material and methods

We retrospectively assessed the electronic dossiers of patients with definite or suspected IFIs who had more than one FDG-PET/CT. The requirement for informed consent for this retrospective study was waived by the local institutional review board (UMCG research register number 201600073). Baseline FDG-PET/CT was defined as an FDG-PET/CT performed before or within 2 weeks of the initiation of antifungal therapy. Patients with a definite diagnosis of IFI had fungi cultured at the beginning or during the course of their treatment. Patients who were diagnosed as clinical IFI had a clinical suspicion of IFI with or without positive serological markers for IFIs, and showed improvement on antifungal treatment and clinical follow-up. For treatment evaluation, FDG-PET/CT was only performed when the treating clinician felt the need to evaluate the IFI with imaging. We included patients who had at least two FDG-PET/CT scans (one at baseline, and one or more during treatment). In each patient included in the study, two or more FDG-PET/CT scans at different time points (one at baseline, and one or more during treatment) were performed. The study was conducted at the University Medical Center Groningen, the Netherlands, and the period covered was from October 2009 until March 2018. This time period was chosen since in October 2009 a PET/CT camera system was installed at our center. Patients with no baseline FDG-PET/CT scan, more than 2 weeks interruption of their antifungal therapy between the serial FDG-PET/CT scans, and no lesion on their baseline scans, such as those with only blood-borne IFIs with no tissue localization, were excluded.

### Review of medical records

The following data were retrieved from the electronical patient files: (1) the microbiology (serology, microscopy, and culture) and pathology (biopsy or surgical excised lesion) that resulted in the identification of IFI, (2) any other imaging that was performed within 2 weeks of each FDG-PET/CT, (3) any procedure that was performed as a result of the FDG-PET/CT scan, (4) start date and duration of antifungal therapy, (5) changes in treatment, (6) whether the patient was dead or alive at the time of data collection, and (7) date and cause of death were documented. We also reviewed the computer-based patient dossiers to determine what decision had been made based on the FDG-PET/CT scan results.

### FDG-PET/CT acquisition for IFIs

All scans were performed according to the EANM procedure guideline for FDG-PET/CT studies [13]. Patients fasted for at least 6 h before the study. The blood glucose was checked to be less than 11 mmol/l before the injection of 3 MBq/kg ^18^F-FDG. PET images were acquired on a research 4 Life (EARL)-accredited integrated PET/CT camera system (Biograph mCT 64 slice PET/CT, Siemens, Knoxville, TN, USA) approximately 1 h after injection of the FDG. Patients were imaged from mid-thigh to vertex of the skull with 3 min per bed position. Low-dose CT was performed for attenuation correction and anatomical localization with the following settings: tube voltage reference 100 kV (adjusted to 80–140Kv as per departmental protocol) with Siemens CarekV switched on, gantry rotation time of 0.5 s, pitch factor of 1, automated exposure control switched on during all acquisitions (Siemens CARE Dose4D), with a quality reference tube-current product of 30mAs (adjusted to 20–50mAs as per departmental protocol). CT images were not enhanced with contrast.

### Analysis of FDG-PET/CT scans

Images were interpreted by experienced nuclear medicine physicians as part of routine clinical care, using Syngo.Via software (Siemens Healthcare, Erlangen Germany). Each scan was re-evaluated by a nuclear physician (AA) who was blinded to the original FDG-PET/CT interpretations. The parameters TLG, MV, SUVmax, and SUVpeak were pre-defined in the Syngo.Via software. For each FDG-PET/CT study, lesions due to IFI were identified, the number of lesions were counted, and volume of interests (VOIs) were drawn around the lesions. IFI lesions were defined as abnormal focal lesions with or without hypometabolic center not related to any procedure or other existing pathology. TLG, MV, SUVmax, and SUVpeak was recorded for every lesion due to IFI for each FDG-PET/CT study. The findings of this second reading by the blinded nuclear physician (AA) were compared to the original FDG-PET/CT reports and any discrepancies were resolved by an independent nuclear physician (AWJMG).

### Analysis of metabolic parameters

The TLG of the individual IFI lesions were summed for each scan to calculate the global TLG for every FDG-PET/CT study. The global MV of the IFI on each scan was also determined by the summation of the MV of the individual lesions. The overall SUVmean of the lFI lesions was calculated by dividing the global TLG by the global MV. The highest SUVmax and SUVpeak for each study were also recorded. SUVmax, SUVpeak, and TLG were corrected for glucose by the formula (parameter × 5/blood glucose in mmol/l) according to the EANM standards.

### Definitions of metabolic response and altered treatment

The responses of IFIs to treatment were classified into three groups based on FDG-PET/CT findings on the final study: (1) patients with a complete metabolic response (CMR), (2) with a partial response, and (3) patients with progression of the infection. CMR was defined as a complete resolution of the FDG uptake due to IFI compared to the background at the site of IFI. A partial response was defined as any reduction in FDG uptake not reaching complete normalisation. Progression of the infection was defined as the appearance of new lesions or an increase in the size or intensity of existing lesions due to IFIs. When FDG-PET/CT led to a cessation or change in antifungal drugs or resulted in surgery, it was defined as alteration of the therapy and having added value. If FDG-PET/CT led to a prolongation of therapy (because of partial response) it was considered as having added value.

### Statistical analysis

Descriptive statistics (mean and standard deviation or mode and range) was used to describe patient and scan data. Data was tested statistically using SPSS Version 23 (IBM Inc., Armonk, NY). Receiver operator characteristic (ROC) analysis was performed to determine whether the metabolic parameters would be able to discriminate patients who had a complete metabolic response (CMR) on their final scan from those who did not have a CMR. Independent *t*-test was used to determine differences in means, and Fisher’s Exact test was used for the difference of categorical values. *P*-values of less than 0.05 were considered significant.

## Results

### Demographic and diagnosis of IFIs and underlying disease

In total, we found 44 patients with IFIs who had more than one FDG-PET/CT. After screening, 28 patients were included. Of the 16 patients that were excluded, eight had no baseline scans, five had their antifungal therapy interrupted by more than 2 weeks in between the serial scans, and in three the baseline scan did not show any FDG-avid lesion (blood-borne IFI in two, and intracerebral candidiasis with no other lesion in one). The demographics of the included patients, type of fungi, diagnosis made, and underlying disease are displayed in Table 1. Seventeen of our patients (61%) were males. The mean age of the patients at the time of their baseline FDG-PET/CT scan was 43 ± 22 years. The diagnosis of IFIs was proven by isolation and growth of fungi in 18 (64%). In the others, diagnosis was made clinically. The majority of patients (*n* = 19, 68%) had a hematologic disorder underlying the IFI. Solid organ transplant was present in six (21%) of the patients. The median duration of treatment from the start of antifungal treatment to the date of the last FDG-PET/CT performed was 33.5 weeks (range 5–242).

**Table 1:** Demographic details of patients, IFIs, and underlying disorders associated with IFI

Patients	
Total (*n*)	28
Female sex: *n* (%)	11 (39%)
Age in years (mean ± SD)	43 ± 22
Type of IFI (*n*)	
Aspergillosis	18 (64%)
*– Aspergillus fumigatus*	8
*– Aspergillus nidus*	1
*– Aspergillus ustus*	1
– Aspergillosis (species not specified)	8
Candida	9 (32%)
*– Candida albicans*	5
*– Candida glabatara*	1
*– Candida dubliniensis*	1
– Candidiasis (unspecified species)	2
Other	1 (4%)
*– Hormografiella aspergillata*	1
Final diagnosis of IFI (*n*)	
Proven	18 (64%)
Clinical	10 (36%)
Risk factor or underlying disorder for IFI (*n*)	
Hematological malignancy or disorder	19 (68%)
– Acute myeloid leukemia	8
– Acute lymphoblastic leukemia	5
– Hematopoietic stem cell transplant	1
– Non-Hodgkin’s lymphoma	3
– Chronic lymphocytic lymphoma	1
– Langerhans cell histiocytosis	1
Solid organ transplant	6 (21%)
– Kidney	1
– Lung	3
– Heart and lung	2
Immunosuppression unrelated malignancy	2 (7%)
– Panniculitis	1
– Rheumatoid arthritis	1
Other	1 (3%)
– Autosomal dominant polycystic kidney disease	1

### FDG PET/CT findings

The findings from the FDG-PET/CT studies are summarized in Table 2. A total of 98 PET/CT studies from 28 patients were analysed. The median number of scans per patient was 3 (range, 2–9).

**Table 2:** Findings of FDG-PET/CT, therapy outcome and change in therapy by fungi, and response outcome on the final study

FDG-PET/CT scans	
Total number reviewed	98
Number of scans per patient: median (range)	3 (2–9)
Duration of therapy till the last PET/CT scan in weeks: median (range)	33.5 (5–242)
Finding on final FDG-PET/CT scan of patients (*n*)	
Complete metabolic response (CMR)	19 (68%)
Partial response (PR)	7 (25%)
Progressive disease (PD)	2 (7%)
Total	28
FDG-PET/CT leading to a change in antifungal	
Fungi type (*n*)	
Aspergillosis	6 (33% of patients with aspergillosis)
Candidiasis	1 (11% of patients with candidiasis)
*Hormografiella aspergillata*	1
Total	8 (29% of all patients)
FDG-PET/CT leading to prolongation of therapy	
Fungi type (*n*)	
Mold	10 (52.6% of pts. with mold)
Yeast	8 (89% of pts. with yeast)
Total	18 (64% of all patients); in four also led to a change, in two also led to stopping of the antifungal
FDG-PET/CT added value	
Change	8 (29% of the total)
Stopped therapy only	6 (21% of total)
Prolongation only	12 (43% of total)
Total	26 (93% of the total patients)

#### Patients with a complete metabolic response

**Fig. 1:**
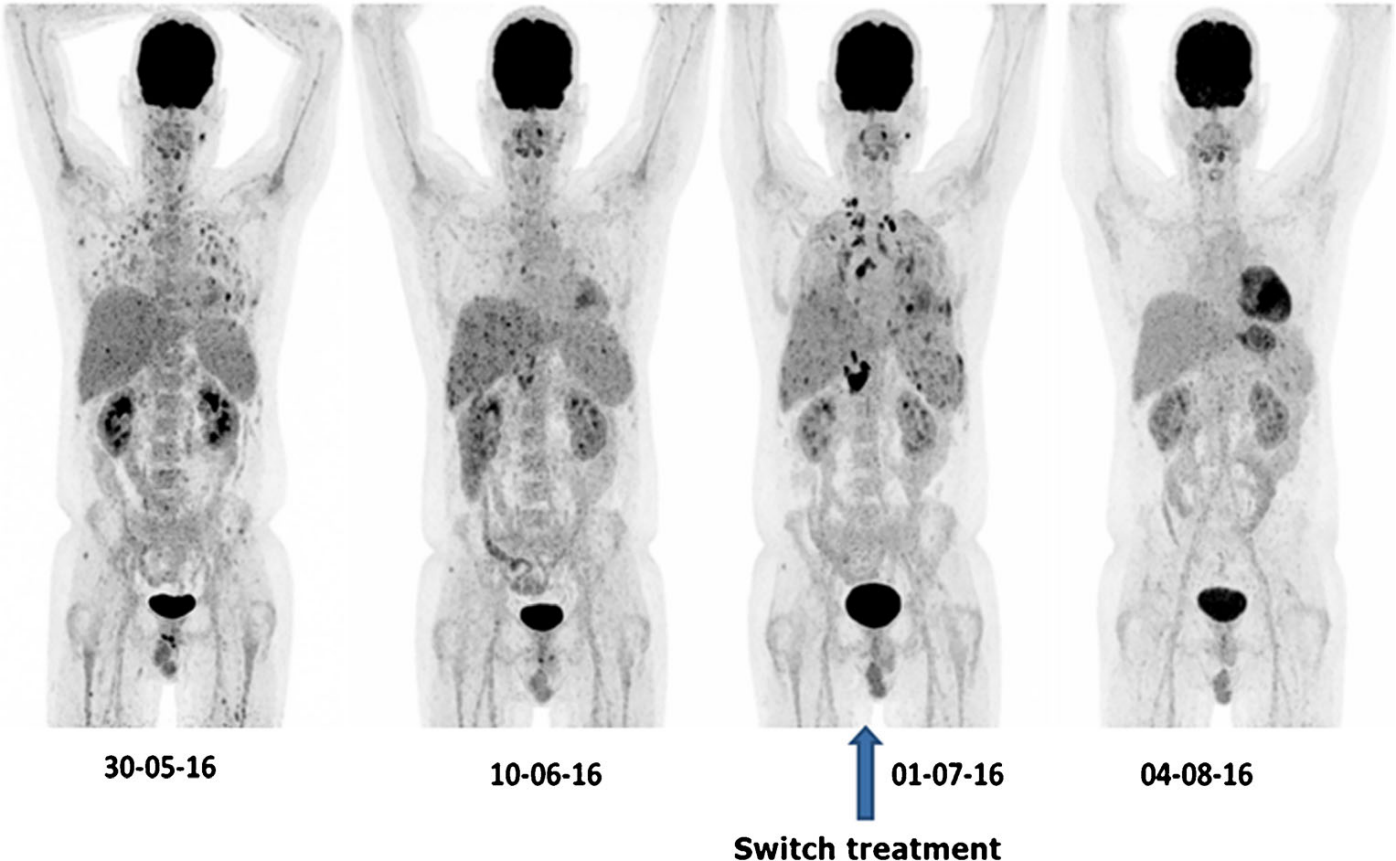
FDG-PET MIP images of a 38-year-old male with acute lymphoblastic leukemia (ALL) who was first thought to have a bacterial infection but was unresponsive to antibiotics. A clinical diagnosis of invasive candidiasis was made, at baseline FDG-PET/CT a global TLG of 401. Follow-up FDG-PET/CT showed a good response with a TLG of 30. Then the patient developed fever, with negative blood cultures, and a repeated FDG-PET/CT showed new lesions with a global TLG of 900. The antifungal therapy was modified, and the patient had a complete metabolic response at the last scan

Nineteen (68%) of the 28 patients showed a CMR at the last FDG-PET/CT scan. In eight of the patients with CMR, FDG-PET/CT led to a cessation of therapy. Figure 1 shows the FDG-PET images of a patient who had a CMR at final scan. In four of the 19 patients (21%), a heterogeneous response to the antifungal therapy was found, with some lesions responding to antifungal therapy but also new lesions appearing.

#### Patients with an incomplete response (partial response and progressive disease)

**Fig. 2:**
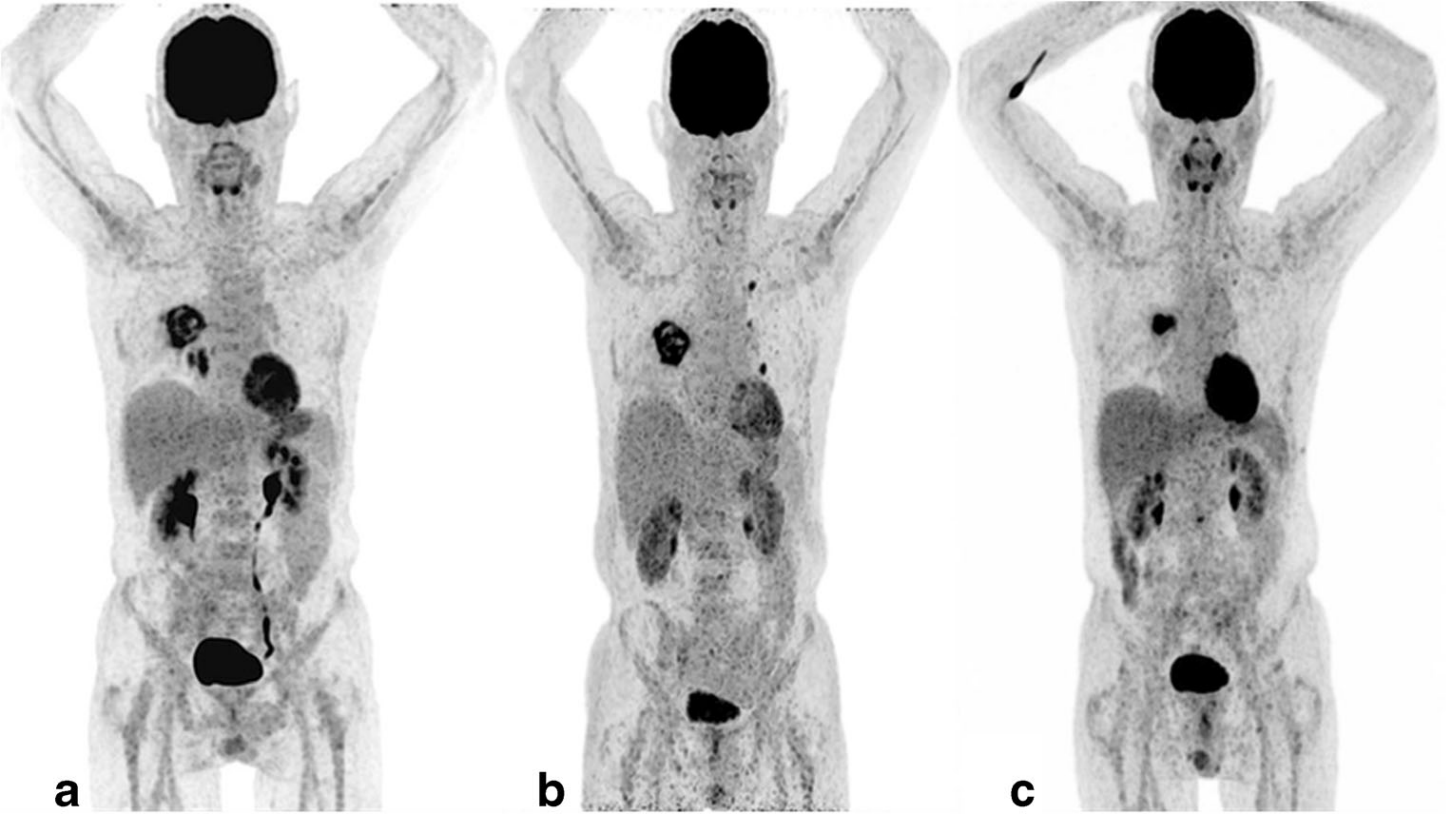
FDG-PET MIP images of a 65-year-old male with AML and diagnosed with aspergillosis from the culture of bronchoalveolar lavage washing (*Aspergillus fumigatus*). Note the complex large heterogeneous pulmonary lesion which did respond but not completely disappear at the final FDG-PET/CT. The patient had a baseline TLG of 144 of the pulmonary lesions. The follow-up scan shows a heterogeneous response, with resolution of the lesions in the right lower lobe below the primary lesion but with appearance of new lesions in the left lung and global TLG of 187. Antifungal therapy was modified, and the last scan showed resolution of lesions except for the primary aspergillus lesion with TLG of 44 after 6 months. This patient had a video-assisted resection of the lesion and subsequently had allogeneic stem cell transplantation (ASCT)

In total, nine patients did not achieve a CMR at the last FDG-PET/CT study. An overview of the results of these patients is shown in Table 3. Two of these patients showed progressive disease, and seven had a partial response at the last FDG-PET/CT study. Two of the patients with a partial response had a complex pulmonary lesion with no other site of IFI at the last FDG-PET/CT scan. Both of the patients had a video-assisted thoracic resection and subsequently underwent autologous stem-cell transplantation. In one of these, the resected tissue did not show any fungal elements, while the second one had fungal elements. The median percentage change in TLG in all the nine patients with a partial response was −80%, ranging from −43% to −90%. Seven out of the patients who did not achieve CMR had aspergillosis and two had candidiasis. Six out of the seven patients with aspergillosis that did not have a CMR response on their last FDG-PET/CT study had residual pulmonary lesions, with most of these lesions being complex with an irregular metabolic uptake. Figure 2 shows the FDG-PET MIP images of a patient with aspergillosis and a residual pulmonary lesion. The two cases due to candidiasis occurred in regions with high FDG signal (brain and kidneys), and were eventually followed up with other anatomically based methods.

**Table 3:** Characteristics of patients who did not have a complete metabolic response on the last FDG-PET/CT scan

ID no.	Age (years)/sex	No. of lesions on baseline	No. of scans done	Tx/weeks	Fungi type	Underlying condition	% change in TLG	Outcome after last PET/CT	Comment
6	2/F	15	6	42	Can^a^	AML^b^	− 44	Therapy prolonged but subsequent follow up with MRI	Brain lesions not distinct on PET/CT, partial response in spleen and kidneys
7	65/M	1	2	26	Asp^c^	AML	− 86	Patient had video-assisted thoracic surgery and FDG avid lesions resected	ASCT^d^ successful with no complication
9	53/F	1	3	13	Can	ADPKD^e^	− 75	Therapy prolonged but subsequent follow up was by clinical parameters	Infected renal cyst, FDG tracer excretion interfered with follow-up by PET/CT
14	3/F	8	3	105	Asp	LCH^f^	+ 25	Therapy changed, but the patient died 14 weeks later	Death due to IFI complications
15	62/M	3	2	23	Asp	NHL^g^	− 80	Treatment prolonged but died	Death due to due to recurrent NHL which could not be treated due to the poor condition of the patient
17	66/M	3	8	43	Asp	AML	− 90	Treatment prolonged but died after 4 weeks	Death related to IFI complication
22	62/M	1	2	13	Asp	ALL^h^	+ 50	Therapy changed: patient died 3 weeks later	Death due to bacterial complications
24	25/F	2	3	22	Asp	ALL	− 62	Still on therapy as time of data collection	Therapy prolonged due to PET/CT
28	65/M	5	3	10	Asp	AML	− 69	Patient had video-assisted thoracic surgery and FDG avid lesions resected	ASCT successfully done with no complication

^a^Can — *Candida sp*.

^b^AML — acute myeloid leukemia

^c^Asp — *Aspergillus sp*.

^d^ASCT — allogeneic stem cell transplantation

^e^ADPKD — autosomal dominant polycystic kidney disease

^f^LCH — Langerhans cell histiocytosis

^g^Non-Hodgkin’s lymphoma

^h^ALL — Acute lymphoblastic leukemia

### Therapy decision-making based on FDG-PET/CT results

#### Prolongation of therapy

FDG-PET/CT resulted in a prolongation in therapy in 18 patients (64%). In these patients, there was still evidence of metabolic activity at the site of the original IFI lesions at a time when patients were clinically stable. This resulted in the prolongation of antifungal therapy. At a later scan, four of these patients also had their therapy altered, and in two patients therapy was stopped. Figure 1 shows four FDG-PET MIP images of a patient that led to both a prolongation and a change of the antifungal therapy.

#### Change in therapy

FDG-PET/CT resulted in a change in therapy in eight patients. The changes in the metabolic parameters over time in one of these patients (the same patient as in Fig. 1), is illustrated in Table 4. The metabolic changes and data for all the eight patients in whom FDG-PET/CT led to a therapy change is available as supplementary material. The global TLG provided information about extent and severity of disease which corresponded to reports. The other metabolic parameters, especially the highest SUVmax, SUVpeak, and SUVmean, severely underestimated (or overestimated) the changes in IFI burden determined by TLG (Table 4 and supplementary material).

**Table 4:** Metabolic parameters and the % change from the previous FDG-PET/CT study of patient whose MIP images are demonstrated in Fig. 1

	1st scan	2nd scan	3rd scan (therapy changed)	4th scan
Global TLG	401.14	29.98 (− 93%)	900.44 (+ 2903%)	No lesion
Global MV	197.61	13.5 (− 93%)	407.44 (+ 2918%)	No lesion
Global SUVmean	2.03	2.22 (+ 9%)	2.21 (0%)	No lesion
Highest SUVmax	7.14	6.43 (− 10%)	18.75 (+ 192%)	No lesion
Highest SUVpeak	4.47	4.61 (+ 3%)	12.35 (+ 168%)	No lesion

#### Added value of PET/CT and alteration

In total, FDG-PET/CT added value to treatment in 26 (93%) of our patients. In 12 it led to a prolongation of therapy only; in six other patients in whom it led to a prolongation, it also led to either a cessation in therapy or change of therapy. In eight patients, it led to a change in antifungal drugs; four of these also had their therapy prolonged at a later FDG-PET/CT scan. In eight it led to cessation of therapy; two of these also had their antifungal drugs changed, leaving six patients who had a cessation in therapy alone (Table 2). FDG-PET/CT altered the management of antifungal treatment (stopped therapy or led to a change) in 14 (50%) of the patients.

### Mortality

Seven of the patients (25%) had died at the time of the analysis. Three of the deaths were in patients who had a CMR to therapy and four in patients with an incomplete response to treatment. The patients with CMR all died more than 6 months after the last PET/CT, while the deaths in patients who did not achieve a CMR occurred within 4 months. The deaths in patients who had CMR occurred at 26, 29, and 30 weeks after the last FDG-PET/CT scan; the cause of death was bacterial complications in all these patients. Three of the deaths were due to the IFI, and all occurred in the patients with an incomplete metabolic response. These deaths occurred at 3, 4, and 13 weeks after the last FDG-PET/CT scan. The last patient who died in the group who did not get a CMR died of recurrent non-Hodgkin’s lymphoma; this patient died 9 weeks after FDG-PET/CT. The difference in overall mortality was not significant (*p* = 0.165) but was significant for mortality due to IFI (*p* = 0.026) using Fisher’s Exact test analysis.

### Differences between baseline FDG-PET/CT findings in CMR and non-responders

A significant difference was found between the average number of lesions for the CMR group and those who did not have a complete response at the final FDG-PET/CT (20.7 vs 4.33, *p* = 0.007). Using receiver operating acharacteristic (ROC) analysis, we found that baseline TLG and MV were able to discriminate between patients eventually having a CMR and patients who did not achieve CMR (Table 4). Baseline TLG had the highest area under the curve (AUC) of 0.95, and discriminated CMR from non-responders at a cut-off of 160 with a sensitivity of 94% and specificity of 100% (Table 5). None of the baseline metabolic parameters (TLG, MV, SUVmax, SUVpeak, and SUVmean) was able to discriminate between patients who eventually needed prolongation of therapy and patients who required a change in treatment based on FDG-PET/CT findings.

**Table 5:** ROC analysis of initial or baseline metabolic parameters and response to therapy

Parameter	AUC^a^	*P* value	Best cut-off	Sensitivity (%)	Specificity (%)
TLG	0.954	< 0.001	160	94	100
MV	0.908	0.001	60	84	75
SUVmax	0.629	0.269	5.47	82	50
SUVpeak	0.576	0.514	4.42	82	40
SUVmean	0.588	0.426	2.35	76	50

^a^AUC — area under the curve

## Discussion

IFI is a life-threatening condition, and only scarce data are available with regard to imaging. Our study provides important data on the role of FDG-PET/CT in monitoring IFIs. Of particular importance is the ability of TLG to provide quantification of the burden of disease due to IFI, the ability of the baseline TLG to predict patients who will achieve a CMR when patients are treated with antifungal drugs, and the fact that FDG-PET/CT has added value in therapy decision-making in a large proportion of the patients.

Our study is the first study to evaluate the role of TLG and MV in infectious diseases. We found TLG was able to provide a quantitative measurement of IFI burden, which correlates well with the reports which were based on visual analysis. TLG and MV were found to be able to predict whether a patient will achieve CMR. This was not the case for the SUVmax, SUVpeak, and SUVmean. This finding indirectly supports the idea that either TLG or MV may be a better parameter to use when comparing scans of patients with systemic diseases such as IFIs compared to SUV parameters. The global TLG at baseline may be used to predict patients who will not completely respond to an antifungal agent and will have persistent FDG uptake or will require surgical resection of IFI lesion. This distinction is essential to avoid unnecessary delay in instituting further therapies such as allogeneic stem cell transplantation (ASCT) based on when FDG-PET/CT is used for monitoring IFIs. While a CMR is achievable in the majority of cases, in some patients this may not always be possible. This may result in unnecessary prolongation of antifungal therapy, with associated side-effects and costs. Furthermore, it may delay further necessary intervention such as ASCT if the monitoring was based on FDG-PET/CT only in these patients. This persistent FDG activity in IFIs has been documented by other authors [14], and has also been reported in other nonfungal infections after patients have been cured of infection [15]. The persistent uptake may be related to old lesions such as fibrosis that the body is trying to eradicate by activated immune cells such as macrophages, giant cells, and lymphocytes with FDG uptake not due to active IFI.

Our findings on the added value of FDG-PET/CT in therapy decision-making in patients with IFIs are in support with other studies that demonstrated the value of FDG-PET/CT in monitoring treatment of IFIs. In one study, FDG-PET/CT was compared to conventional CT in detecting and guiding management of IFIs. This study found 94% (30/32) of the FDG-PET/CT scans to be useful in evaluating therapy response [16]. In another study, FDG-PET/CT was found to lead to altered treatment by stopping or reducing the dose in 15% (8/54) or changing or increasing the dose of antifungal drugs in 31% (17/54) cases [17]. IFI is a rare disease, and a large multi-centre study will be necessary to validate our findings.

In patients with underlying hematologic malignancies who develop IFI after neutropenia, antifungal therapy is usually necessary for an extensive period of time (months to even years) as the immune system of the host is not effective in clearing the organism. If the treatment of the underlying condition of the IFI involves further depression of the immune system such as ASCT, there is the risk of dissemination of IFI during treatment. Monitoring is crucial to treat IFIs adequately, but also not to subject the patients to unnecessary treatment with toxic effects and to lower the expensive costs of the treatment [2]. In our study, the mean duration of treatment till the last FDG-PET/CT was 33.5 weeks (range, 5–242). International organizations such as the European Society of Clinical Microbiology and Infectious Diseases (ESCMID), the Infectious Diseases Society of America (IDSA), and the European Conference on Infection in Leukaemia recommend a search for disseminated infection in a leukemic patient with an IFI recovering from neutropenia [18]. The methods they recommended for excluding a disseminated IFI included transoesophageal echocardiography, funduscopic evaluation, and/or abdominal imaging. These methods are region- or organ-specific, and are not able to detect occult IFIs at other sites concurrently. FDG-PET/CT as a whole body tool is able to evaluate several sites in one study.

Organs in the body that have a high metabolic signal may limit the role of FDG-PET/CT as a monitoring tool [4]. FDG-PET/CT monitoring of IFIs was not optimal in patients with predominantly cerebral and renal lesions in our study. The advantages and disadvantages of the various imaging tools must be appreciated by the clinician, and the most appropriate utilized for the best patient outcome.

The serial FDG-PET/CT scans for IFI monitoring were acquired at different time points during the treatment. This is because the patients with IFIs have different underlying risk factors with different degrees of immune suppression. Furthermore, the genus and species of fungi causing IFIs are also different, making it difficult to perform FDG-PET/CT at a fixed point in time. The timing of the follow-up scan should be dictated by the clinical condition of the patient, allowing personalized therapy decisions of this life-threatening infection.

The mortality in our patients directly due to IFIs was 11%, and the overall mortality was 25%. All the patients who died due to IFIs were patients who did not have a CMR at the last scan. The mortality of 11% of IFI patients who were on treatment underscores the importance of developing new strategies for the management of IFIs. New strategies including vaccine, cytokiness, and cellular immunotherapy have been investigated at a preclinical level [19]. FDG-PET/CT is well positioned to provide quantitative assessment of the burden of IFI in patients when these therapeutic interventions are translated to humans. Furthermore, the lack of statistical significance between the mortality of the CMR group and non-CMR group, but the significant difference when mortality due to IFI is considered, may suggest a prognostic role of metabolic response on the last study. However, our sample size is too small to fully explore this. Larger multicentre prospective studies are needed to confirm the prognostic value of FDG-PET/CT study in monitoring IFIs.

Our study has some limitations regarding the small sample size, the retrospective nature of our study, and data coming from a single center, which may limit its generalization. Our strict inclusion criteria may have systematically excluded very ill patients who were unable to undergo several FDG-PET/CT scans. In some of our patients the IFIs were not proven by biopsy, but this is in our opinion the clinical practice; it is not always possible to have full proof by biopsies.

## Conclusion

FDG-PET/CT is useful for therapy monitoring in patients with IFIs, and helps in therapy decision-making, resulting in alteration of treatment strategy in 50% of patients. TLG and MV at baseline scan were found to be useful predictors of metabolic response to antifungal therapy. Since IFIs are uncommon, prospective multicentre trials in homogenous patient populations are necessary to confirm these findings. Eventually, FDG-PET/CT may be added to the diagnostic and therapeutic flowcharts of patients with IFI.

## Electronic supplementary material

ESM 1 (DOCX 29 kb)
