# Supplementary material for: Role of FDG PET/CT in monitoring treatment response in patients with invasive fungal infections
Source: Eur J Nucl Med Mol Imaging. 2018 Oct 21;46(1):174–83. doi: 10.1007/s00259-018-4192-z (PMC6267682; doi:10.1007/s00259-018-4192-z)
Supplement: Supplementary file 1 — (DOCX 29 kb) [file 259_2018_4192_MOESM1_ESM.docx]

Metabolic parameters and % change in serial FDG PET/CT in patients in whom therapy was changed

Supplementary Table 1 (Patient 2 in the study, same as Table 4 and represent change for Fig 1) .

|  | 1^st^ scan | 2^nd^ scan | 3^rd^ scan* | 4^th^ scan |
| --- | --- | --- | --- | --- |
| Global TLG | 401.14 | 29.98 (-93%) | 900.44 (+2903%) | No lesion seen |
| Global MV | 197.61 | 13.5 (-93%) | 407.44 (+2918%) | No lesion seen |
| Global SUVmean | 2.03 | 2,22 (+9%) | 2.21 (0%) | No lesion seen |
| Highest SUVmax | 7.14 | 6.43 (-10%) | 18.75 (+192%) | No lesion seen |
| Highest SUVpeak | 4.47 | 4.61 (+3%) | 12.35 (+168%) | No lesion seen |

*- FDG PET/CT led to a switch in therapy

Supplementary Table 2 (Patient 4 in the study)

|  | 1^st^ scan | 2^nd^ scan | 3^rd^ scan* | 4^th^ scan* | 5^th^ scan |
| --- | --- | --- | --- | --- | --- |
| Global TLG | 253.1 | 103.86 (-59%) | 167.28 (+61%) | 218.16 (+30%) | No lesion seen |
| Global MV | 68.56 | 30.2 (-56%) | 41.74 (+38%) | 104.65 (+151%) | No lesion seen |
| Global SUVmean | 3.69 | 3.44 (-7%) | 4.01 (+17%) | 2.08 (+48%) | No lesion seen |
| Highest SUVmax | 6.94 | 6.76 (-3%) | 7.67 (+13%) | 4.06 (-47%) | No lesion seen |
| Highest SUVpeak | 5.21 | 4.62 (-11%) | 5.11 (-11%) | 3.12 (-64%) | No lesion seen |

*- FDG PET/CT study led to a switch in therapy

Supplementary Table 3 (Patient 14 in the study)

|  | 1^st^ scan | 2^nd^ scan | 3^rd^ scan* |
| --- | --- | --- | --- |
| Global TLG | 155.2 | 97.68 (-37%) | 422 (+332%) |
| Global MV | 66.4 | 17.94 (-73%) | 97.4 (+443%) |
| Global SUVmean | 2.34 | 5.44 (+132%) | 4.33 (-20%) |
| Highest SUVmax | 7.97 | 6.59 (-17%) | 7.89 (+20%) |
| Highest SUVpeak | 5.92 | 4.32 (-27%) | 6.55 (+34%) |

*- FDG PET/CT study led to a switch in therapy

Supplementary Table 4 (Patient 22 in the study)

|  | 1^st^ scan | 2^nd^ scan* |
| --- | --- | --- |
| Global TLG | 118.26 | 279.64 (+137%) |
| Global MV | 57.55 | 44.09 (24%) |
| Global SUVmean | 2.05 | 6.34 (+209%) |
| Highest SUVmax | 3.87 | 16.52 (+327%) |
| Highest SUVpeak | 2.98 | 12.34 (+314%) |

*- FDG PET/CT study led to a switch in therapy

Supplementary Table 5 (Patient 28 in the study same as patient represented by Fig 2 in article)

|  | 1^st^ scan | 2^nd^ scan* | 3^rd^ scan |
| --- | --- | --- | --- |
| Global TLG | 143.59 | 186.68(+30%) | 44.85 (-76%) |
| Global MV | 36.92 | 48.99(+33%) | 8.61 (-82%) |
| Global SUVmean | 3.89 | 3.81(-21%) | 5.21 (+37%) |
| Highest SUVmax | 7.89 | 9.84 (+25%) | 9.04 (-8%) |
| Highest SUVpeak | 6.44 | 6.91(+7%) | 6.86 (-1%) |

*- FDG PET/CT study led to a switch in therapy

Supplementary Table 6 (Patient 6 in the study)

|  | 1^st^ scan | 2^nd^ scan* | 3^rd^ scan | 4^th^ scan | 5^th^ scan* | 6^th^ scan |
| --- | --- | --- | --- | --- | --- | --- |
| Global TLG | 122,61 | 1578,73 (+1188%) | 409,53  (-74%) | 87,23  (-79%) | 97,11 (+11%) | 69,02  (-29%) |
| Global MV | 85,65 | 437,7  (+411%) | 176,39  (-60%) | 35,78  (-78%) | 45  (+26%) | 39,33  (-13%) |
| Global SUVmean | 1,43 | 3,6  (+152%) | 2,32  (-36%) | 2,44  (+5%) | 2,16  (-10%) | 1,75  (-19%) |
| Highest SUVmax | 5.84 | 7,45  (+28%) | 4,87  (-35%) | 4,59  (-6%) | 4,44  (-3%) | 3,23  (-27%) |
| Highest SUVpeak | 4.71 | 5.31  (13%) | 3,82 (-28%) | 4,57 (+20%) | 3,15  (31%) | 2,88 (9%) |

*- FDG PET/CT study led to a therapy switch

Supplementary Table 7 (patient 19 in the study)

|  | 1^st^ scan | 2^nd^ scan | 3^rd^ scan* | 4^th^ scan* | 5^th^ scan* | 6^th^ scan | 7^th^ scan | 8^th^ scan | 9^th^ scan |
| --- | --- | --- | --- | --- | --- | --- | --- | --- | --- |
| Global TLG | 422,96 | 229,23 (-46%) | 346,62 (+51%) | 593,98 (+71%) | 323,55  (-45%) | 51,7  (-84%) | 15,55  (70%) | 7,67  (51%) | No lesion |
| Global MV | 179,07 | 58,24  (-67%) | 133,33  (+129%) | 275,95  (+107%) | 180,37  (-35%) | 24,43  (-86%) | 7,46  (69%) | 5,51  (26%) | No lesion |
| Global SUVmean | 2,36 | 3,94  (+67%) | 2,6  (-34%) | 2,15  (-17%) | 1,79  (-17%) | 2,12  (+18%) | 2,08  (-2%) | 1,39  (50%) | No lesion |
| Highest SUVmax | 13,69 | 13,31  (-3%) | 14,33  (+8%) | 12,59  (-12%) | 4,31  (-66%) | 5,29  (+21%) | 3,65  (31%) | 2,09  (43%) | No lesion |
| Highest SUVpeak | 9.87 | 10.11  (+2%) | 9.01  (-11%) | 8.78  (-3%) | 3.68  (-58%) | 3.71  (+1%) | 2.41  (35%) | 1.76  (27%) | No lesion |

*- FDG PET/CT study leading to a therapy switch

Supplementary Table 8 (Patient 17 of the study)

|  | 1^st^ scan | 2^nd^ scan | 3^rd^ scan* | 4^th^ scan | 5^th^ scan* | 6^th^ scan | 7^th^ scan | 8^th^ scan |
| --- | --- | --- | --- | --- | --- | --- | --- | --- |
| Global TLG | 884,38 | 786,44  (-11%) | 942,42  (+20%) | 608,84  (-35%) | 640,95  (+5%) | 319.57  (-50%) | 33.33  (-90%) | No lesion  seen |
| Global MV | 437,08 | 198,58  (-55%) | 216,64  (+9%) | 287,55  (+33%) | 180,83  (-37%) | 91,36  (-49%) | 11,54  (-87%) | No lesion  seen |
| Global SUVmean | 2,02 | 3,96  (+96%) | 4,35  (+10%) | 2,12  (-51%) | 3,54  (+67%) | 3,5  (1%) | 2,89  (-17%) | No lesion seen |
| Highest SUVmax | 5,78 | 8,54  (+48%) | 11,12  (+30%) | 6,92  (-35%) | 10,44  (+50%) | 9,07  (-13%) | 6.05  (-33%) | No lesion seen |
| Highest SUVpeak | 5.08 | 7.67  (+51%) | 9.54  (+24%) | 5  (-48%) | 7.27  (+45%) | 6.47  (-11%) | 4.3  (-49%) | No lesion seen |

*- FDG PET/CT study led to a therapy switch

Comment

In all eight patients, the highest SUVmax, SUVmean, and SUV mean markedly underestimated or overestimated the percentage change of disease burden as estimated by the global TLG. The changes in global MV gave the best approximation of the changes but in some cases showed marked differences. The changes in highest SUVmean gave the worst approximation of all the metabolic parameters.
